# Effects of scale worm parasitism on interactions between the symbiotic gill microbiome and gene regulation in deep sea mussel hosts

**DOI:** 10.3389/fmicb.2022.940766

**Published:** 2022-08-15

**Authors:** Gaoyou Yao, Hua Zhang, Panpan Xiong, Huixia Jia, Maoxian He

**Affiliations:** ^1^CAS Key Laboratory of Tropical Marine Bio-resources and Ecology, Guangdong Provincial Key Laboratory of Applied Marine Biology, South China Sea Institute of Oceanology, Chinese Academy of Sciences, Guangzhou, China; ^2^Southern Marine Science and Engineering Guangdong Laboratory (Guangzhou), Guangzhou, China; ^3^College of Marine Science, University of Chinese Academy of Sciences, Beijing, China; ^4^Institution of South China Sea Ecology and Environmental Engineering, Chinese Academy of Sciences, Guangzhou, China

**Keywords:** Haima cold seep, scale worm, deep sea mussel, parasitism, host-microorganism interactions, gene expression

## Abstract

Diverse adaptations to the challenging deep sea environment are expected to be found across all deep sea organisms. Scale worms *Branchipolynoe pettiboneae* are believed to adapt to the deep sea environment by parasitizing deep sea mussels; this biotic interaction is one of most known in the deep sea chemosynthetic ecosystem. However, the mechanisms underlying the effects of scale worm parasitism on hosts are unclear. Previous studies have revealed that the microbiota plays an important role in host adaptability. Here, we compared gill-microbiota, gene expression and host-microorganism interactions in a group of deep sea mussels (*Gigantidas haimaensis*) parasitized by scale worm (PA group) and a no parasitic control group (NPA group). The symbiotic microorganism diversity of the PA group significantly decreased than NPA group, while the relative abundance of chemoautotrophic symbiotic bacteria that provide the host with organic carbon compounds significantly increased in PA. Interestingly, RNA-seq revealed that *G. haimaensis* hosts responded to *B. pettiboneaei* parasitism through significant upregulation of protein and lipid anabolism related genes, and that this parasitism may enhance host mussel nutrient anabolism but inhibit the host’s ability to absorb nutrients, thus potentially helping the parasite obtain nutrients from the host. In an integrated analysis of the interactions between changes in the microbiota and host gene dysregulation, we found an agreement between the microbiota and transcriptomic responses to *B. pettiboneaei* parasitism. Together, our findings provide new insights into the effects of parasite scale worms on changes in symbiotic bacteria and gene expression in deep sea mussel hosts. We explored the potential role of host-microorganism interactions between scale worms and deep sea mussels, and revealed the mechanisms through which scale worm parasitism affects hosts in deep sea chemosynthetic ecosystem.

## Introduction

Deep sea cold seeps are among the most extreme environments on Earth, owing to the high hydrostatic pressure, poor oxygenation, and toxicity of the ecosystem (Feng et al., 2018). Although these extreme environmental factors are unfavorable for living organisms, specialized deep sea species are continually being discovered and create cold seep communities (Levin et al., 2016). The main feature of the deep sea cold seep ecosystem supporting this biomass is chemosynthetic production, wherein chemoautotrophic microorganisms perform redox reactions to produce energy for carbon fixation (Van Dover et al., 2002). Thus, the adaptive mechanisms of benthic communities in these extreme environments have attracted substantial scientific interest (Gaudron et al., 2017; Sun et al., 2017).

Diverse adaptations to the challenging deep sea environment are expected to be found across all deep sea organisms. For instance, compared with shallow water mussels (*Modiolus philippinarum*), deep sea mussels (*Gigantidas platifrons*) show greater removal of toxic substances from cells and stabilization of protein structures, thus, indicating adaptation to extremely toxic conditions (Sun et al., 2017). In deep sea chemosynthetic ecosystems, scale worms are thought to adapt to the deep sea by parasitizing mussels in deep sea chemosynthetic ecosystems (Zhang et al., 2017). A total of 71.5% of *Bathymodiolus azoricus* deep sea mussels in Lucky Strike have been found to be parasitized by *Branchipolynoe seepensis* (Britayev et al., 2003). Furthermore, the parasitism rate exceeds 90% in another deep sea mussel, *G. haimaensis*, according to our findings (unpublished data). These parasites live within the pallial cavity of the mussel mantle in tunnel-like structures formed by the external demibranches and gill filaments, within which they adjust their position according to their feeding behavior (Kádár et al., 2006; Britayev et al., 2007; Plouviez et al., 2008). The biotic interaction between deep-sea mussels and scale worms is most known in a chemosynthetic ecosystem (Bebianno et al., 2018). However, the mechanisms underlying the effects of scale worm parasitism on hosts is unclear.

The microbiota has been found to play a crucial role in host adaptation in previous studies (Kokou et al., 2018; Butt and Volkoff, 2019; Schoeler and Caesar, 2019). In particular, the gills of the deep sea mussel are symbiotic with chemotrophic bacteria, which play key roles in nutrient metabolism and sulfur adaptation of hosts (Sun et al., 2017). The host organism and its associated microorganisms are often described as a single entity, the holobiont, because they are so closely linked (Larsson et al., 2012; Levy et al., 2015). Therefore, the microbiota of the host is expected to also undergo similar changes when the host adapts to stressful conditions. Considering the hologenome concept, we hypothesized that parasitism by scale worms might affect host deep sea mussel associated gill microbial species, as well as the response of these microorganisms to scale worm parasitism.

Here, we compared the gene expression (*via* RNA-seq) and microbiota (*via* 16S rRNA gene sequencing) in a group of deep sea mussels with scale worms (PA group) and a no parasitic control group (NPA group). The correlations between host gill microbiota data and gene expression were identified through integrative analysis, which allowed us to characterize potential interactions between host microorganisms and genes, thus, providing insights into the mechanisms of scale worm parasitism in hosts.

## Materials and methods

### Sample collection

**Figure 1:**
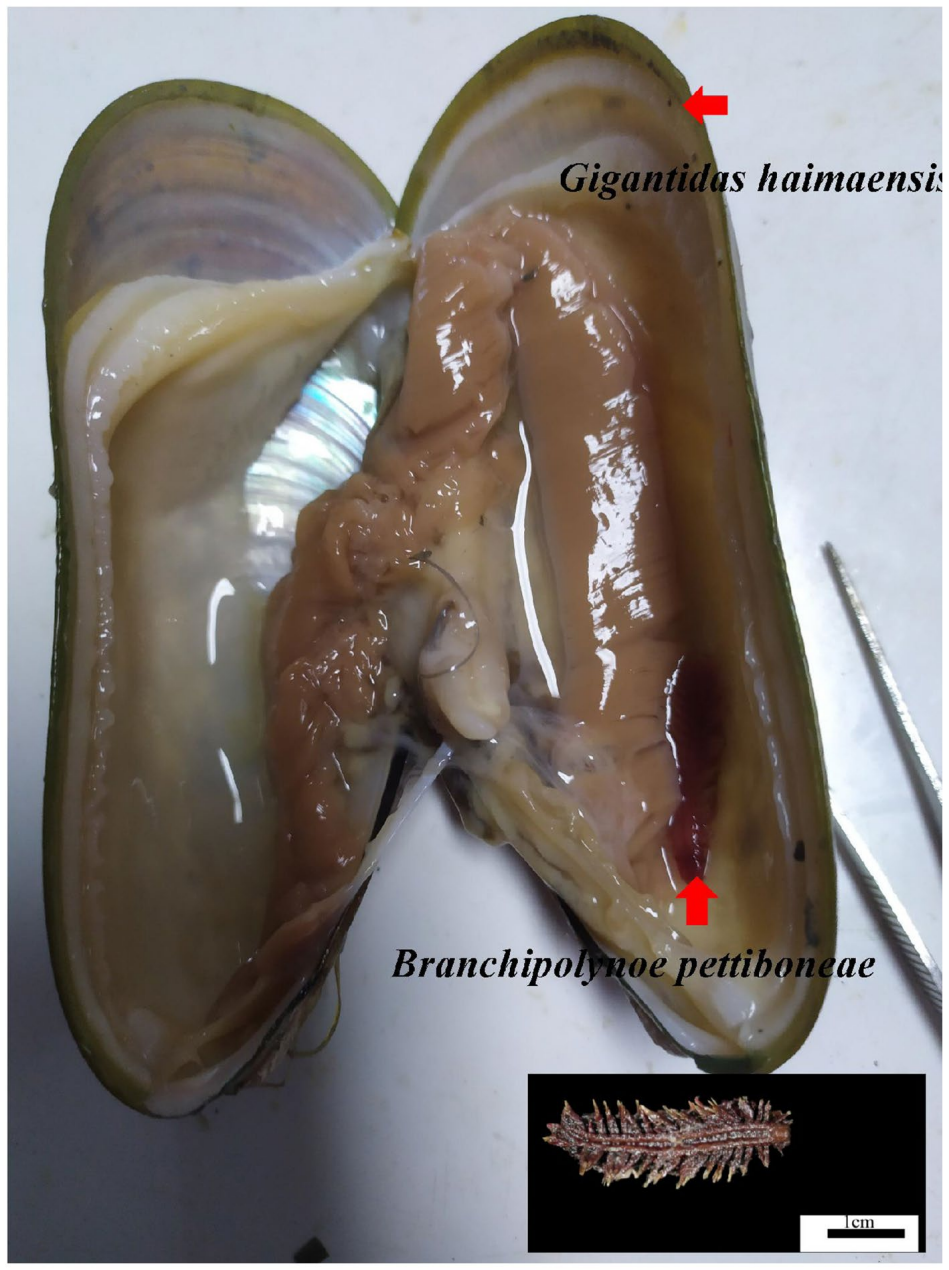
Photograph of the scale worm *Branchipolynoe pettiboneae* and its host deep sea mussel *Gigantidas haimaensis.* Shell length of the *G. haimaensis* is 8.2 cm.

During the HYDZ6-202005 cruise of the *Haiyang 6* research vessel of the Guangzhou Marine Geological Survey, deep sea *G. haimaensis* mussels were collected with the submersible ROV *Haima* in the Haima cold seep (depth 1,435 m) in September, 2020. The mussels were dissected to detect the presence of scale worms (Figure 1). Finally, six samples of mussels parasitized by scale worms (PA group) and six unparasitized mussel samples (NPA group) were obtained. The gills of the mussels were sampled and stored in liquid nitrogen.

### 16S rRNA gene sequencing

DNA from the gills was extracted with the CTAB method (Thakuria et al., 2009). The quality and concentration of the extracted DNA were detected on 1% agarose gels. DNA was diluted with sterile water to l μg/μL. The primers (341/F: CCTAYGGGRBGCASCAG, 806R: GGACTACNNGGGTATCTAAT) was used to amplify the 16S rRNA genes (V3–V4; Xu et al., 2019). A total of 15 μl (10 ng template DNA and 0.2 μM of forward and reverse primers) of Phusion® High-Fidelity PCR Master Mix (New England Biolabs) was used for all PCR reactions. Thermal cycling included an initial denaturation at 98°C for 1 min; 30 cycles of denaturation at 98°C for 10 s, annealing at 50°C for 30 s, and elongation at 72°C for 30 s; and a final step at 72°C for 5 min. The loading buffer (containing SYB green) was mixed with the PCR products and electrophoresed on 2% agarose gels for detection. The PCR products were mixed in equal proportions and then purified with a Qiagen Gel Extraction Kit (Qiagen, Germany). As recommended by the manufacturer, sequencing libraries were generated with a TruSeq® DNA PCR-Free Sample Preparation Kit (Illumina, United States), and index codes were added. A Qubit 2.0 Fluorometer (Thermo Scientific) and Agilent Bioanalyzer 2,100 system were used to assess library quality. The Illumina platform was used to sequence the library and generate 250 bp paired-end reads.

### Microbiota data processing, quality assessment, and diversity analysis

The paired-end reads were assigned to samples according to their unique barcodes and then truncated to remove the barcode and primer sequence. FLASH (VI.2.7^1^) was used to merge paired-end reads (Magoč and Salzberg, 2011). Quality filtering of raw tags was conducted under specific filtering conditions to produce high-quality clean tags according to the QIIME. V1.9.1^2^ quality control process (Caporaso et al., 2010; Bokulich et al., 2013). To detect chimeric sequences, we compared the tags with a reference database (Silva database^3^) by using the UCHIME algorithm (Edgar et al., 2011). The chimera sequences were then removed (Haas et al., 2011), and the effective tags were finally obtained. With Uparse software v7.0.1001, all sequences with >97% similarity were assigned to the same OTUs (Edgar, 2013). According to the 16S rRNA gene reference SSUrRNA database, taxonomy was assigned with Mothur. QIIME was used to calculate Shannon’s alpha diversity index.

### Host RNA extraction, sequencing, quality control, and filtering

TRIzol (Invitrogen, United States) was used to obtain total RNA from the gills, and a Bioanalyzer 2,100 system Assay Kit (Agilent Technologies, CA, United States) was used to assess RNA integrity. RNA sample preparations were made from a total of 1 μg RNA per sample. In brief, poly-T oligonucleotide-conjugated magnetic beads were used to purify mRNA from total RNA. Divalent cations were used for fragmentation under elevated temperatures in First Strand Synthesis Reaction Buffer (5×). First strand cDNA was synthesized with M-MuLV reverse transcriptase (RNase H) and random hexamer primers. The second strand cDNA was subsequently synthesized with RNase H and DNA polymerase I. Exonuclease/polymerase were used to convert the remaining overhangs into blunt ends. To prepare for hybridization, adapters with hairpin loop structures were ligated after adenylation of the 3’ends of the cDNA fragments. The library fragments were purified with an AMPure XP system (Beckman Coulter, Beverly, United States) to select cDNA fragments 370 × 420 bp in length. PCR was then conducted with Index (X) Primer, universal PCR primers, and Phusion High-Fidelity DNA polymerase. Finally, PCR products were purified with an AMPure XP system, and library quality was evaluated with an Agilent Bioanalyzer 2100 system equipped with a Qubit 2.0 Fluorometer. According to the manufacturer’s instructions, the index-coded samples were clustered on a cBot Cluster Generation System with a TruSeq PE Cluster Kit v3-cBot-HS (Illumina). On the Illumina platform, 150 bp paired-end reads were generated. In-house scripts were used to process the fastq format raw data. After removal of poly-N reads, adapter reads, and low-quality reads from the raw data in this step, clean data (clean reads) were obtained. Additionally, the Q20 and Q30 of the clean data were calculated. Clean, high-quality data were used for all downstream analyses. The transcriptome was assembled with Trinity (Grabherr et al., 2011).

### Host RNA-seq differential expression and enrichment

The DESeq2 R package (1.20.0) was used for differential expression analysis (Love et al., 2014). Benjamini and Hochberg’s approach to controlling the false discovery rate was used to adjust *p*-values. The differentially expressed genes (DEGs) were identified with DESeq2 had|log_2_ (fold change, FC)| > 1&padj <0.05. The databases Clusters of Orthologous Groups of proteins (COG), Eukaryotic Orthologous Groups of proteins (KOG), Non-Redundant Protein Sequence Database (NR) and Swiss-Prot (manually annotated and reviewed protein sequences) were used to annotate the functions of DEGs. With the clusterProfiler R package, we analyzed DEGs in Kyoto Encyclopedia of Genes and Genomes (KEGG) pathways to determine statistical enrichment.

### Integrated analysis of interactions between host genes dysregulation and changes in the microbiota

We performed correlation analysis between host gene expression data for all DEGs and gill microbiota abundance data (relative abundance) for the top 30 taxa (genus level). In this analysis, we used Spearman correlation, because it performs better with normalized counts (gene expression) and compositional data than other metrics such as Pearson correlation (Weiss et al., 2016).

## Results

The 16S rRNA sequencing yielded a total of 11 million PE reads, with approximately 987,000 PE reads per sample. An average of 68,495 raw tags were obtained and filtered, thus, generating 61,091 effective tags per sample. The Q30 ranged from 90.24 to 93.3%, with an average of 91.69% (Supplementary Table S1). A total of 3,210 OTUs were isolated from two groups (PA and NPA), and 392 shared OTUs were identified between PA and NPA (Supplementary Figure S1). The Venn diagram provided as Supplementary Figure S1 illustrates that the two samples (NPA.1 and PA.1) in their treatment group possessed much OTUs than the closest samples. To examine if the principal conclusions are affected by the mussels samples (PA1 and NPA1), we revalidated the results of microbes diversity, genes expression patterns and integrated analysis after removing NPA1 and PA1. The comparative analysis suggested that the sample NPA1 and PA1 may not affect the results for the primary outcome in our study, which is in close agreement with our conclusion. The comparative results were included in the Supplementary Materials 2.

**Figure 2:**
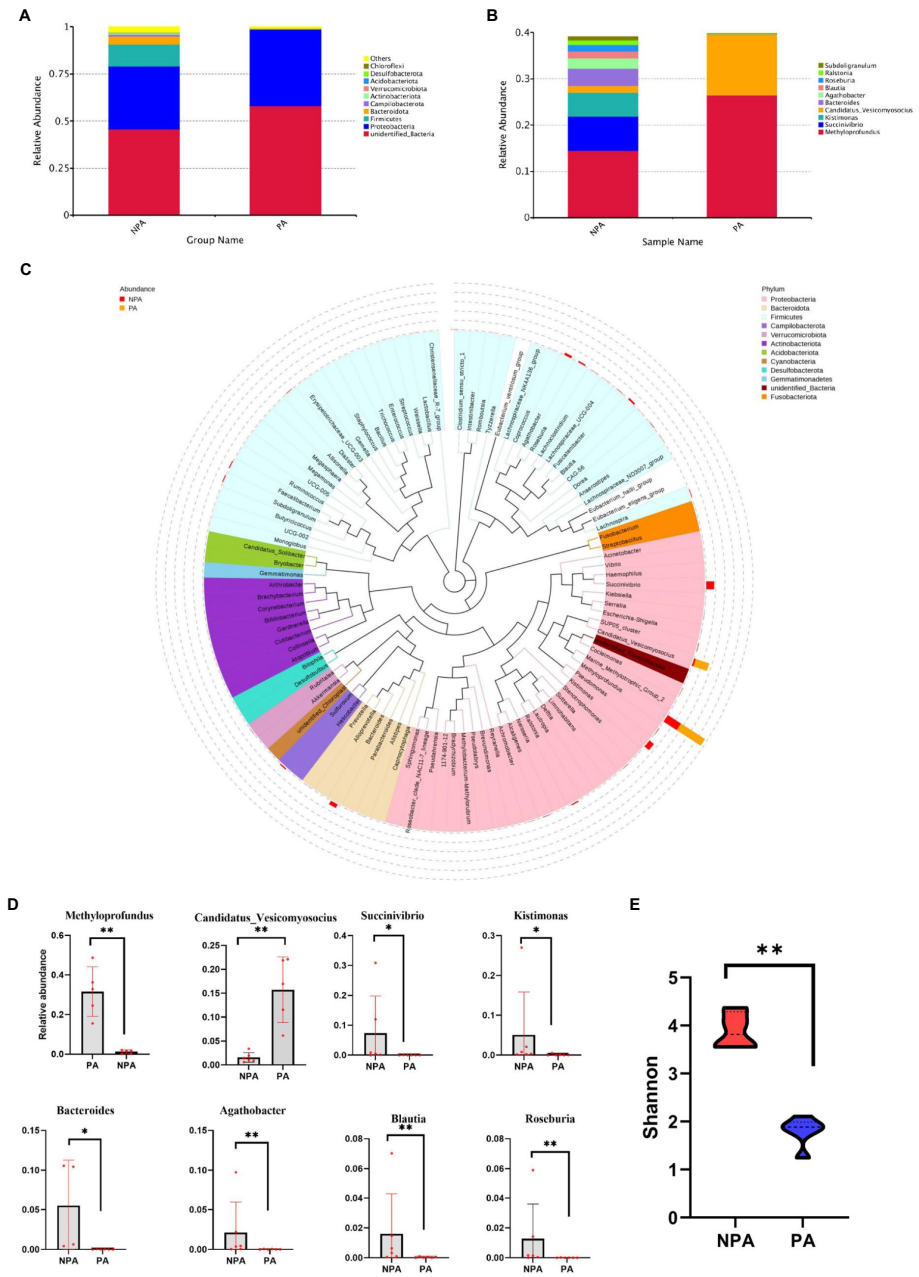
Microbial community structure and diversity between NAP and PA. **(A)** Relative bacterial abundance at phylum level in NA and NPA. **(B)** The all microbial community structure in PA and NPA at genus level. **(C)** Comparison of relative abundance at top 10 genus level between groups PA and NPA. **(D)** Statistical analysis showing the most significant changes between groups PA and NPA, *mean *p* < 0.05 and **mean *p* < 0.01. **(E)** The Shannon diversity in NPA and PA.

Regarding the community structure of PA and NPA, the Shannon diversity index of PA markedly decreased (*p* < 0.01, Figure 2E). The core OTU of PA (13) was lower than that of NPA (48, Supplementary Figure S2). At the phylum level, in PA, the relative abundance of Proteobacteria (*p* = 0.0079) significantly increased, whereas that of Bacteroidetes (*p* = 0.0087), Actinobacteriota (*p* = 0.0152) and Firmicutes (*p* = 0.0260) significantly decreased than NPA (Figure 2A). At the genus level, in PA, the relative abundance of *Methyloprofundus* (*p* = 0.0079) and *Candidatus_Vesicomyosocius* (*p* = 0.0043) significantly increased, whereas that of *Succinivibrio* (*p* = 0.0281), *Kistimonas* (*p* = 0.0152), *Bacteroides* (*p* = 0.0095), *Agathobacter* (*p* = 0.0087), and *Blautia* (*p* = 0.0043) significantly decreased than NPA (Figures 2B–D).

A total of 267,278,813 raw reads were generated from the 12 gill samples RNA-seq of *G. haimaensis*. After removal of adaptor sequences, low-quality sequences, and ambiguous nucleotides, a total of 77.58 Gb quality sequences were yielded by paired-end sequencing of *G. haimaensis,* ranging from 5.9 Gb to 6.83 Gb per sample, with a *Q20* higher than 97% in all 12 samples. The error rate of the sequencing data was 0.03% (Supplementary Table S2). Clean data were assembled into 151,518 transcripts with an average length of 969 bp and an N50 length of 1,315 bp. The longest transcript of each gene was selected, and 69,197 unigenes were obtained, with an average length of 952 bp and an N50 length of 1,291 bp. The length distribution and range of all transcripts and unigenes are shown in Supplementary Figure S3. All the results indicated that good quality sequence data were obtained. Based on GO analysis, all genes were linked to 953 biological processes, 188 cellular components, and 180 molecular functions annotated for GO term assignments, mainly related to cellular processes, binding and metabolic processes (Supplementary Figure S6). All genes were associated with 291 KEGG annotations, and were mainly linked to signal transduction, transport and catabolism, endocrine system and immune system (Supplementary Figure S7).

**Figure 3:**
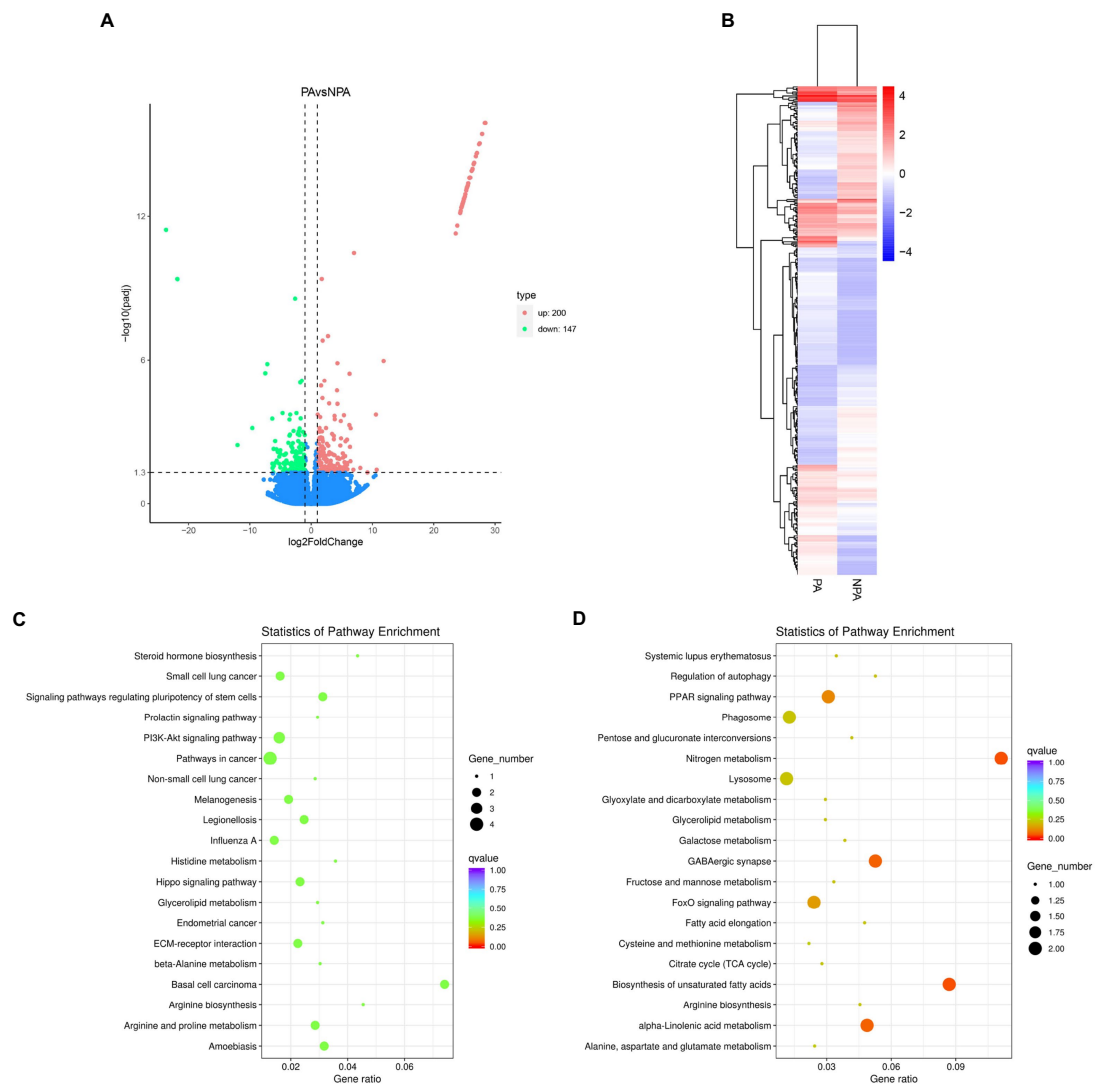
Differentially expressed genes (DEGs) in gill of deep-sea mussels *G. haimaensis* transcriptome between NPA and PA. **(A)** Volcano plot displaying DEGs between NPA and PA group. The green dots and red dots represent downregulated and upregulated DEGs, respectively. A total of 347 unigenes were identified as differentially expressed. **(B)** Hierarchical clustering analysis for the differentially express genes between PA and NPA transcriptomes. The horizontal lines represent the expression pattern of each gene, and the vertical rows represent PA and NPA samples. The expression level is represented by color intensities (red color indicates the higher expression, and blue color indicates the lower expression of the gene). **(C)** Downregulated DEGs enriched in KEGG pathways. **(D)** KEGG enrichment analysis of annotated DEGs, Upregulated DEGs enriched in KEGG pathways.

To examine the functions of the unigenes, we annotated 43,704 unigenes (63.15%) according to various databases (NR, SwissProt, PFAM, NT, KO and KOG). The database with the most hits (32,214 unigenes, 46.55%) was the GO database (Supplementary Figure S4; Supplementary Table S3). The best hit of most *E*-value distributions and the annotated unigenes of the matched sequences are shown in Supplementary Figure S5. In analysis of differential gene expression, we identified 347 unigenes differentially expressed between PA and NPA, of which 147 and 200 unigenes were down-regulated and up-regulated, respectively (Figure 3A). PA and NPA had two distinct profiles represented by the results of heatmap analysis (Figure 3B). KEGG enrichment analysis indicated that upregulated DEGs (PA vs. NPA) were most enriched in the function “nitrogen metabolism in metabolism” (*p* = 0.0012) and “biosynthesis of unsaturated fatty acids” (*p* = 0.0019; Figure 3D). According to KEGG enrichment analysis, downregulated DEGs were most enriched in “basal cell carcinoma,” followed by “amoebiasis,” and “signaling pathways regulating pluripotency of stem cells” (Figure 3C).

**Figure 4:**
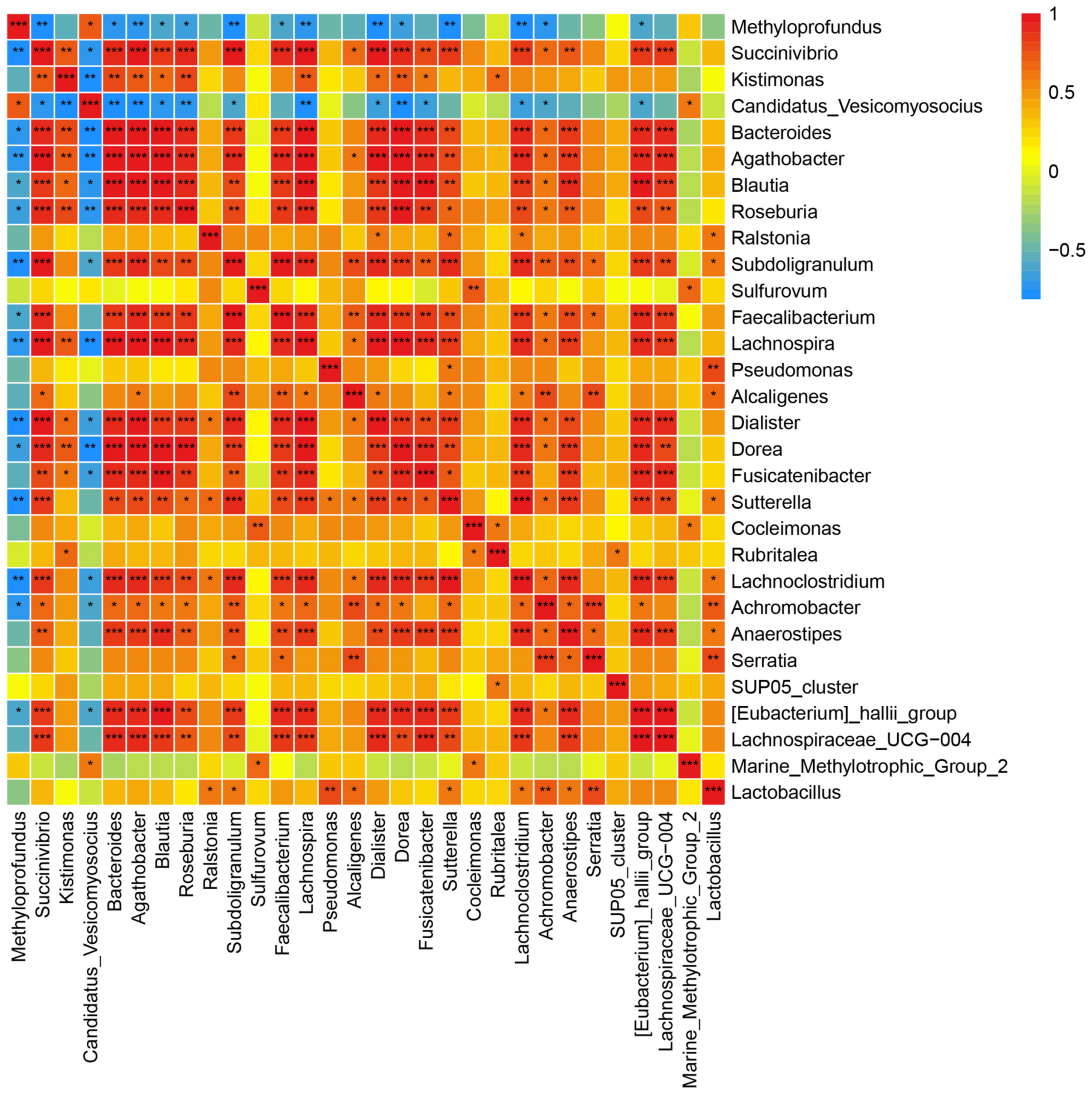
Correlation plot depicting microbe-microbe correlations. Color indicate the magnitude of the correlation, asterisks indicate significance of correlation (*** indicates *p* value <0.001 ** indicates *q* value <0.01 and *indicates *q* value <0.05).

Spearman correlation was used to infer the relationship between DEGs and microbial communities. A significant negative correlation was observed between *Candidatus Vesicomyosocius* and *Methyloprofundus*, two major chemosynthetic symbiotic bacteria in *G. haimaensis*, and most other gill-associated microorganisms (Figure 4; Supplementary Table S5). A total of 10,410 pairs (30 × 347) were explored, and the relationships between the top 347 DEGs and top 30 gill-associated microorganisms at the genus level are shown in Figure 4; Supplementary Table S6. In particular, we found several significantly positive gene-taxa correlations between *Candidatus Vesicomyosocius* and Cluster-29 (NR description: glutamine synthetase, e-value = 1.3E-158, FC = 27.8; Spearman r = 0.815, *p* = 0.001), and *Methyloprofundus* and Cluster-205.17402 (NR: adenosine kinase 1-like, e-value = 1.1E-147, FC = 1.6592, *p* = 8.63E-06; Spearman *r* = 0.762, *p* = 0.006). A clear significantly negative correlation (Spearman *r* = −0.689, *p* = 0.0132) was observed between *Candidatus Vesicomyosocius* and Cluster-205.21036 (NR: solute carrier family 23 member 1-like, *e*-value = 3.1E-137, FC = −5.4458, *p* = 1.65E-05).

## Discussion

Analysis of the PA and NPA revealed distinct differences between microbiota. We observed a significant increase in *Candidatus Vesicomyosocius* in PA (PA relative abundance = 32.8%, NPA relative abundance = 1.63%, *p* = 0.0079) and *Methyloprofundus* (PA relative abundance = 16.9%, NPA relative abundance = 1.25%, *p* = 0.0043). The chemoautotrophic bacteria are considered symbionts that fix carbon dioxide, detoxify hydrogen sulfide, and provide their hosts with organic carbon compounds (Wang et al., 2021). Endosymbiotic relationships with chemosynthetic bacteria have enabled many deep-sea invertebrates to flourish in hydrothermal vents and cold seeps (Ip et al., 2021). The increased abundance of chemoautotrophic microbiota in PA may indicate the host’s increased demand due to *B. pettiboneaei* parasitism. Interestingly, the sulfur-oxidizing bacterium *SUP05*, another chemoautotrophic bacterium (Dede et al., 2022), decreased significantly in PA (*p* = 0.0256). This finding may be associated with the deep sea mussel in cold seep ecosystem’s reliance on methane rather than sulfide for its primary productivity.

The health of the host depends on microbiota stability and the presence of specific bacteria (Coryell et al., 2018). In this study, the gill bacterial diversity displayed marked differences in relation to parasite colonization status. The gill bacterial diversity of PA was significantly lower than that in NPA, as indicated by the Shannon index. Comparable results have been reported in other studies. A significant decrease in bacterial diversity and richness in scallop *Argopecten purpuratus* has been detected at 48 h post Vibrio-injected challenge (Muñoz et al., 2019). Previous studies have also indicated that the parasite *Trichuris suis* significantly affects the microbiota diversity in the colons of pigs. Moreover, the diversity and composition of the human gut microbiota has been found to be threatened by *Necator americanus* and other helminth parasites (Naveed and Abdullah, 2021). In PA, host homeostasis was negatively affected by losses and shifts in the core microbiota. Lower microbiota diversity among invertebrates is normally associated with the presence of pathogens, and a lower species richness is typically associated with increased vulnerability to pathogens (Richards et al., 2019). The remarkable loss of *Alistipes* and *Bacteroides*, which have protective effects against diseases (Parker et al., 2020; Wang et al., 2022), may suggest this vulnerability relative to their hosts *G. haimaensis.*

Previous studies concur that *B. pettiboneaei* is parasitic to deep sea mussels and feeds on the host (Takahashi et al., 2012); however, little is known regarding its molecular mechanisms. We identified differentially regulated host genes between the PA group and NPA group through KEGG analysis, functionally enriched for mainly three activities in host, nutrients anabolism, low immune responder and growth inhibitory activity (Supplementary Table S7).

The host *G. haimaensis* responded to *B. pettiboneaei* parasitism through upregulating protein and lipid anabolism related genes. Compared with those in NPA, genes involved in nutrient metabolism pathways (e.g., nitrogen metabolism and arginine biosynthesis), such as *glnA* (FC = 27.8, *p* = 2.89E-21) and *GLUL* (carbonic anhydrase, FC = 8.001, *p* = 0.00015), were significantly upregulated in PA. In addition, the host *G. haimaensis* parasitized by *B. pettiboneaei* displayed significant changes in the expression of genes involved in lipid metabolism, such as *FADS2* (fatty acid desaturase 2, FC = 1.0167, *p* = 7.82E-05). *FADS2* encodes a protein that is involved in the biosynthesis of unsaturated fatty acids and plays an important role in lipid anabolism, as the first enzyme in the biosynthesis of long-chain fatty acid (≥C20; Terova et al., 2021). Similarly, the rate-limiting enzyme diacylglycerol o-acyltransferase in cotton melon aphids parasitized by *Lysiphlebia japonica* has been found to increase by 3.24 fold, and almost all key genes in the glycerolipid synthesis pathway have been found to be up-regulated with respect to the non-parasitic group (Zhang et al., 2015). Parasitism also has been reported to promote host glycolysis (Febvay et al., 1999). Interestingly, solute carrier family 23 member 1-like (*p* = 3.1E-137, FC = −23.679), a key gene controlling the properties and absorption of fatty acids (Gunawan et al., 2018), and Cluster-205.6681 (NR: inactive pancreatic lipase-related protein 1, FC = −1.4587, *p* = 4.38E-05), involved in fat digestion and absorption pathways, were significantly downregulated, whereas a lipid anabolism related gene was significantly up-regulated. We therefore speculated that *B. pettiboneaei* parasitism might enhance the nutrient anabolism of the host *G. haimaensis* but inhibit the ability of the host to absorb nutrients, thus, helping the parasite obtain nutrients from the host. Furthermore,the significant increase in chemoautotrophic symbiotic bacteria in PA also provided additional evidence of increased host nutrient synthesis. This finding supports the hypothesis that the relationship between scale worms and deep-sea mussels is kleptoparasitic, involving interspecific stealing of already procured food (Britayev et al., 2007).

Among the DEGs in PA, we identified enrichment in cancer pathways (e.g., basal cell carcinoma) among the most relevant KEGG pathways, and significant downregulation of growth-related genes such as *Wnt6* (Wnt Family Member 6, FC = −2.019, *p* = 4.18E-05) and *FZD5* (frizzled-5, FC = −1.493, *p* = 0.0001). WNT is a member of the Wnt signaling pathway, and Wnt protein is restricted to the gut region in bivalve oysters (*Crassostrea gigas*), and plays an important role in oyster growth (Liu et al., 2018). FZD proteins function as transmembrane receptors for WNT ligands, and are involved in cell proliferation, DNA damage repair, and stemness (Sun et al., 2020). The significant downregulation of growth-related genes indicated that *B. pettiboneaei* may disfavor the growth of the host *G. haimaensis.* Inhibition of host growth by parasitism has been reported in previous studies. For instance, hymenoptera host mass has been found to significantly decrease within 24 h after parasitism by *Mythimna separata*, regardless of the host instar at parasitism (Chu et al., 2014).

Generally, parasitism triggers a strong immune response in the host. For example, Atlantic salmon (*Salmo salar*) parasitized by the ectoparasite *Neoparamoeba perurans* mounts a local and systemic defense, and its immune responses are upregulated, including the transcription factors znfOZF-like and znf70-like, and their associated gene networks (Botwright et al., 2021). However, the host *G. haimaensis* immune response to *B. pettiboneaei* parasitism was not significant: no significant immune pathways, such as the Toll pathway, which has a major role in innate immunity of bivalves (Lu et al., 2022), were found in PA (Figure 3D). Instead, most of the annotated immune genes were significantly downregulated (Supplementary Table S7). Strategies have arisen during host–parasite co-evolution to alter the interface of parasites and inhibit the host immune response, thus helping many parasites evade their hosts immune system. For instance, Hemomucin from the parasitoid wasp *Macrocentrus cingulum* may protect parasitic embryos from being encapsulated by their hosts, through evading the host immune reactions (Hu et al., 2014). We found that immune related genes—such as *arg* (arginase, FC = −1.9399, *p* = 8.00E-05), which participates in a variety of inflammatory diseases through downregulating nitric oxide synthesis (Munder, 2009)—significantly decreased in PA. This finding may reveal a strategy used by parasites to promote their survival inside hosts by suppressing host *G. haimaensis* immunity.

The microbiota has widespread, modifiable effects on host gene regulation (Richards et al., 2019). In the deep sea scale worm-mussel parasite model, we observed that the host microbiota significantly affects *G. haimaensis* host gene expression: most of the taxa significantly correlated with the gene expression also significantly differed in abundance between PA and NPA. Furthermore, we found an agreement between transcriptomic and microbiota responses to *B. pettiboneaei* parasitism, including a significantly positive correlation between host protein and lipid metabolism gene expression and the relative abundance of chemoautotrophic bacteria. We additionally observed a strong correlation between *Candidatus Vesicomyosocius* and Cluster-298 (NR description: glutamine synthetase, *e*-value = 1.3E-158, FC = 27.8; Spearman *r* = 0.815, *p* = 0.0014), an important enzyme involved in ammonia assimilation (Reitzer, 2009). Another correlation (Spearman *r* = 0.762, *p* = 0.006) was observed between highly expressed Cluster-205.8142 (NR: adenosine kinase 1-like, *e*-value = 1.1E-147, FC = 1.6592, *p* = 8.63E-06) and *Methyloprofundus*, which plays a role in metabolism of adenosine triphosphate; this response may serve to increase lipid and protein synthesis in the host by providing energy for synthesis reactions. A similar correlation between the *Methyloprofundus* and NADP (isocitrate dehydrogenase) that important number of the citrate cycle was also observed. These results indicated that chemoautotrophic symbiotic bacteria affect host gene expression, and the bacterial-gene expression profile can contribute to *B. pettiboneaei* parasitism of the host *G. haimaensis.*

## Conclusion

In summary, we report the effects of scale worm parasitism on interactions between the symbiotic gill microbiome of deep sea mussels and gene regulation. The symbiotic microorganism diversity of PA significantly decreased while the relative abundance of chemoautotrophic symbiotic bacteria, which provide their hosts with organic carbon compounds, significantly increased. The results of RNA-seq revealed that the *G. haimaensis* host responded to *B. pettiboneaei* parasitism through upregulating protein metabolism and lipid metabolism, and that *B. pettiboneaei* parasitism may enhance the nutrient anabolism of the host but inhibit the ability of the host to absorb nutrients, thus, potentially helping the parasite obtain nutrients from the host. We observed an agreement between transcriptomic and microbiota responses to *B. pettiboneaei* parasitism, with a significantly positive correlation between host protein and lipid metabolism gene expression and the relative abundance of chemoautotrophic symbiotic bacteria. Together, our data provide new insights into the effects of parasite scale worms on orchestrated modulation of deep sea mussel host gene expression and changes in symbiotic bacteria.

## Data availability statement

The original contributions presented in the study are publicly available. This data can be found at: https://www.ncbi.nlm.nih.gov/genbank/, PRJNA859434 and PRJNA859410.

## Author contributions

GY performed most of the experiments, analyzed data, and wrote the manuscript. MH planned and designed the research. HZ, PX, and HJ assisted in the experiment. All authors contributed to the article and approved the submitted version.

## Funding

This work was supported by the Basic and Applied Basic Research Project of Guangdong Province (2019B030302004-04) and Key Special Project for the Introduced Talents Team of Southern Marine Science and Guangdong Engineering Laboratory (Guangzhou; GML2019ZD0401).

## Conflict of interest

The authors declare that the research was conducted in the absence of any commercial or financial relationships that could be construed as a potential conflict of interest.

## Publisher’s note

All claims expressed in this article are solely those of the authors and do not necessarily represent those of their affiliated organizations, or those of the publisher, the editors and the reviewers. Any product that may be evaluated in this article, or claim that may be made by its manufacturer, is not guaranteed or endorsed by the publisher.

## Footnotes

^1^ http://ccb.jhu.edu/software/FLASH/

^2^ http://qiime.org/scripts/split libraries_fastq.html

^3^ http://www.arb-silva.de/
