# Corrigendum: Effects of scale worm parasitism on interactions between the symbiotic gill microbiome and gene regulation in deep sea mussel hosts

**DOI:** 10.3389/fmicb.2022.1048145

**Published:** 2022-10-06

**Authors:** Gaoyou Yao, Hua Zhang, Panpan Xiong, Huixia Jia, Maoxian He

**Affiliations:** ^1^CAS Key Laboratory of Tropical Marine Bio-resources and Ecology, Guangdong Provincial Key Laboratory of Applied Marine Biology, South China Sea Institute of Oceanology, Chinese Academy of Sciences, Guangzhou, China; ^2^Southern Marine Science and Engineering Guangdong Laboratory (Guangzhou), Guangzhou, China; ^3^College of Marine Science, University of Chinese Academy of Sciences, Beijing, China; ^4^Institution of South China Sea Ecology and Environmental Engineering, Chinese Academy of Sciences, Guangzhou, China

**Keywords:** Haima cold seep, scale worm, deep sea mussel, parasitism, host-microorganism interactions, gene expression

In the original article, there was an error in the affiliations.

Instead of “Gaoyou Yao^1, 2^, Hua Zhang^1, 3, 4^, Panpan Xiong^1, 2^, Huixia Jia^1, 2^ and Maoxian He^1, 3, 4*^” it should be “Gaoyou Yao^1, 2, 3^, Hua Zhang^1, 2, 4^, Panpan Xiong^1, 3^, Huixia Jia^1, 3^ and Maoxian He^1, 2, 4*^”

^1^ CAS Key Laboratory of Tropical Marine Bio-resources and Ecology, Guangdong Provincial Key Laboratory of Applied Marine Biology, South China Sea Institute of Oceanology, Chinese Academy of Sciences, Guangzhou, China

^2^ Southern Marine Science and Engineering Guangdong Laboratory (Guangzhou), Guangzhou, China

^3^ College of Marine Science, University of Chinese Academy of Sciences, Beijing, China

4 Institution of South China Sea Ecology and Environmental Engineering, Chinese Academy of Sciences, Guangzhou, China

The authors apologize for this error and state that this does not change the scientific conclusions of the article in any way. The original article has been updated.

## Publisher's note

All claims expressed in this article are solely those of the authors and do not necessarily represent those of their affiliated organizations, or those of the publisher, the editors and the reviewers. Any product that may be evaluated in this article, or claim that may be made by its manufacturer, is not guaranteed or endorsed by the publisher.

