# Supplementary material for: Effects of scale worm parasitism on interactions between the symbiotic gill microbiome and gene regulation in deep sea mussel hosts
Source: Front Microbiol. 2022 Aug 15;13:940766. doi: 10.3389/fmicb.2022.940766 (PMC9421265; doi:10.3389/fmicb.2022.940766)
Supplement: Supplementary file 1 [file Presentation_1.PDF]

Supplementary Materials for

Effects of scale worm parasitism on interactions between the symbiotic gill microbiome and gene regulation in deep sea mussel hosts

Gaoyou Yao, Hua Zhang, Panpan Xiong , Huixia Jia and Maoxian He*

*Corresponding author
Prof. Dr. Maoxian He
E-mail: hmx2@scsio.ac.cn

This PDF file includes:

Figures S9 to S13

The Venn diagram provided as Supplemental Figure 1 illustrates that the two samples (NPA.1 and PA1) in their treatment group possessed much OTUs than the closest samples. To examine if the principal conclusions are affected by the mussels samples (PA1 and NPA1),We revalidated the results of microbes diversity,genes expression patterns and integrated analysis after removing NPA1 and PA1.

1.The microbial diversity after removing NPA1 and PA1

The results shown that the relative abundance of chemogenic symbiotic bacteria increased significantly in *G.haimaensis* host after being parasitized by scale worm (Fig S9 and Fig S10). In addition,the microbes diversity were also compared between the new result and our previous result. The microbial diversity in the group PA also showed a significant decrease as in the non-deleted results after the removal of PA1 and NPA1 (Fig S11). The re-reanalysis results agrees to our findings ,with essentially identical result (The relative abundance of chemogenic symbiotic bacteria increased significantly while the gill symbiotic microbes diversity decreased significantly in PA) .

2. Genes expression pattern

For RNA-seq, to test whether the PA1 (one of the six mussel in PA group) and NPA1 (one of the six mussel in NPA group) affect genes expression patterns presented in our manuscript. We compared the genes expression patterns between deleted samples (removing the sample PA1 in PA group and NPA1 in NPA group) and non-deleted samples. 34 differentially expressed genes (nutrients anabolism related genes, immune related genes and growth related genes in Table S6) were selected for the validation. The expression patterns (deleted PA1 and NPA1) exhibited a good consistency with previous results (Fig S12). The positive correlation (R^2 = 0.8724) between the results of deleted samples and non-deleted confirmed that gene expression profiles in our manuscript were reliable.

3. Integrated analysis of interactions between host genes dysregulation and changes in the microbiota

Identical results were also observed in the integrated analysis after removing the sample PA1 and NPA1 from both microbiome and gene expression analysis. For example, the new correlation between *Candidatus Vesicomyosocius* and Cluster-298 was consistent with the correlations presented in our manuscript (r=0.836,p=0.0025 **VS** r=0.815, p=0.0014). The similar phenomenon were also observed in correlation between *Methyloprofundus* and Cluster-205.8142 (r=0.745,p=0.018 **VS** r=0.762, p=0.006). Regarding the correlation among top30 microbiota, the results from reanalysis were consistent with previous analyses, with a significant negative correlation between *Candidatus Vesicomyosocius* and *Methyloprofundus*, two major chemosynthetic symbiotic bacteria, and most other gill-associated microorganisms (Fig 13).

These new results (the re-reanalysis of the microbes diversity,genes expression patterns and integrated analysis) suggested that the sample NPA1 and PA1 may not affect the results for the primary outcome in our study,which is in close agreement with our conclusion. Although the sample NPA1 and PA 1 possessed much OTUs than the other closest samples, the high proportion core microbiome that is essential to the ecology of the microbial community did not change

significantly than other closest samples. This may have partly contributed to the explanation of the re-reanalysis that were consistent with our previous findings after eliminating NPA1 and PA1.

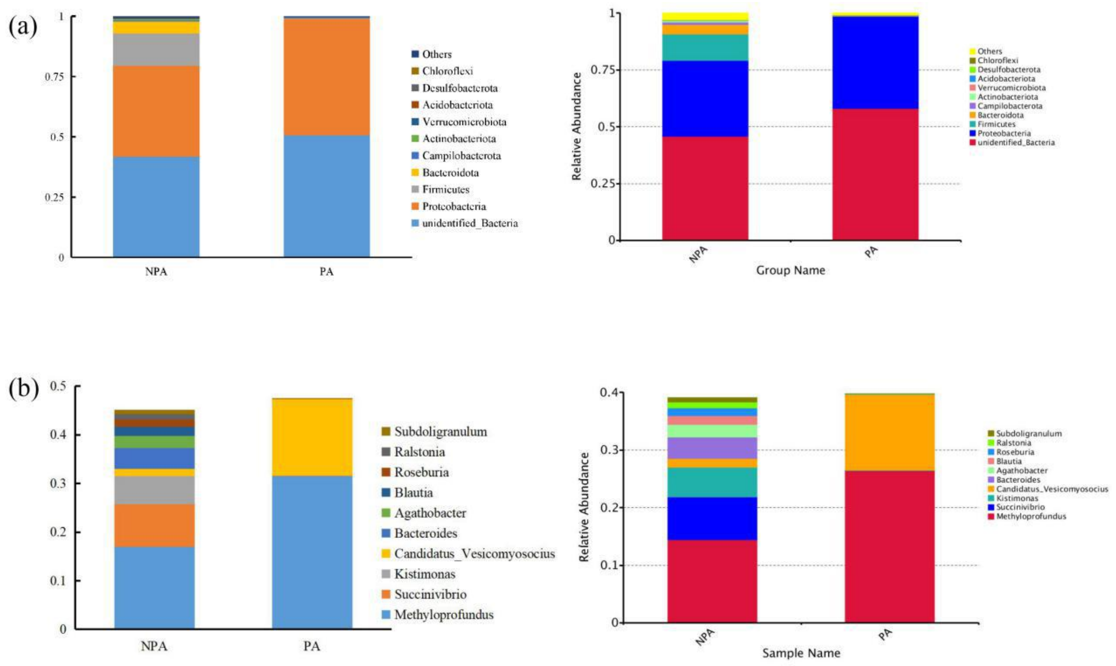

FigS9 Comparison of relative bacterial abundance between non-deleted samples and deleted samples . (a) Left:the relative bacterial abundance at top10 phylum level after removing the sample PA1 and NPA1; right: the relative bacterial abundance presented in our manuscript (the sample PA1 and NPA1 were kept). (b) Left: the relative bacterial abundance at top10 genus level. right: the relative bacterial abundance presented in our manuscript.

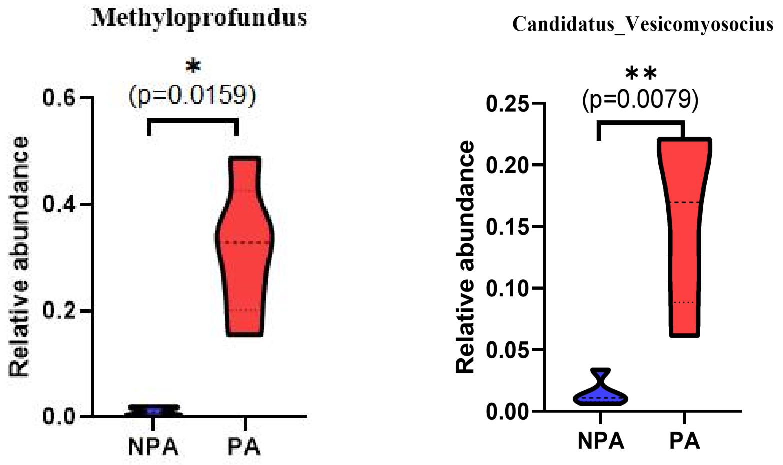

Fig S10 The relative abundance of two major chemosynthetic symbiotic bacteria in *G.haimaensis* (the sample PA1 and NPA1 were removed).

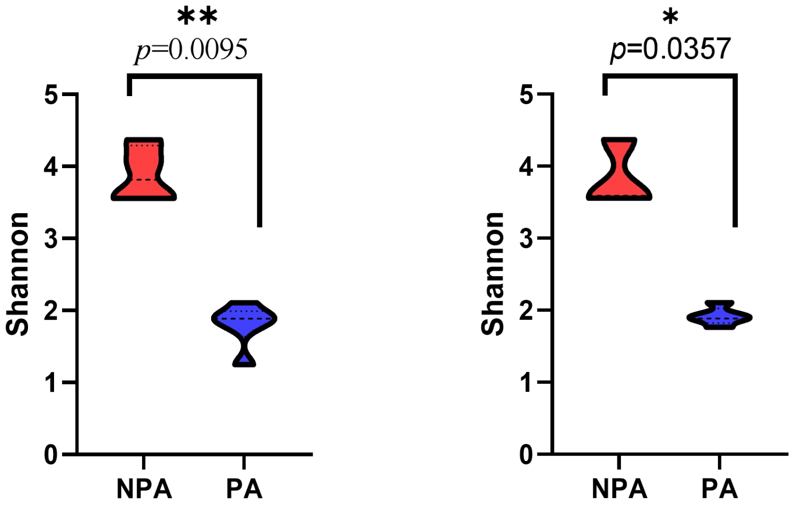

(1) With PA1 and NPA1 (2) Without PA1 and NPA1

Fig S11 Comparison of Shannon index.

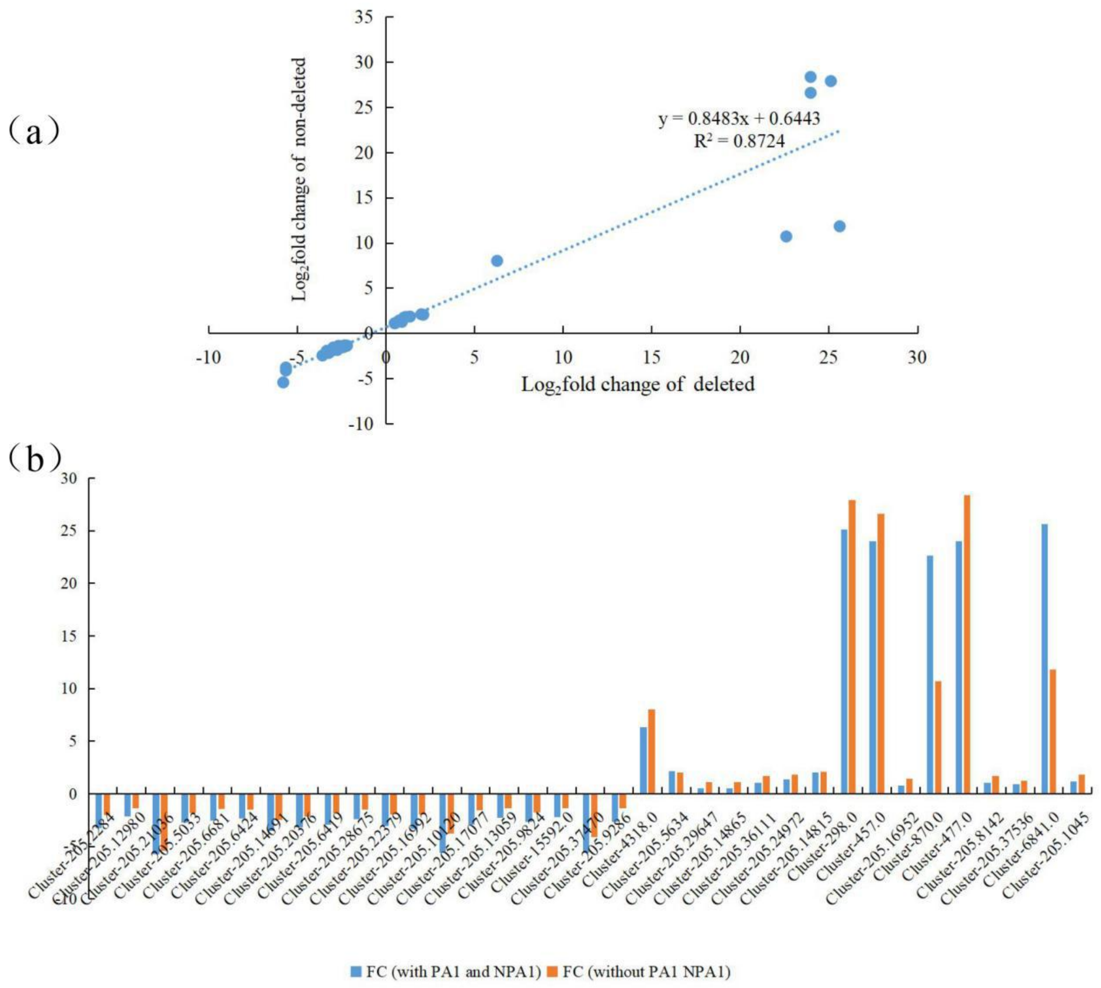

Fig S12 Correlation (a) and comparison (b) of gene expression pattern between non-deleted samples and deleted sample.

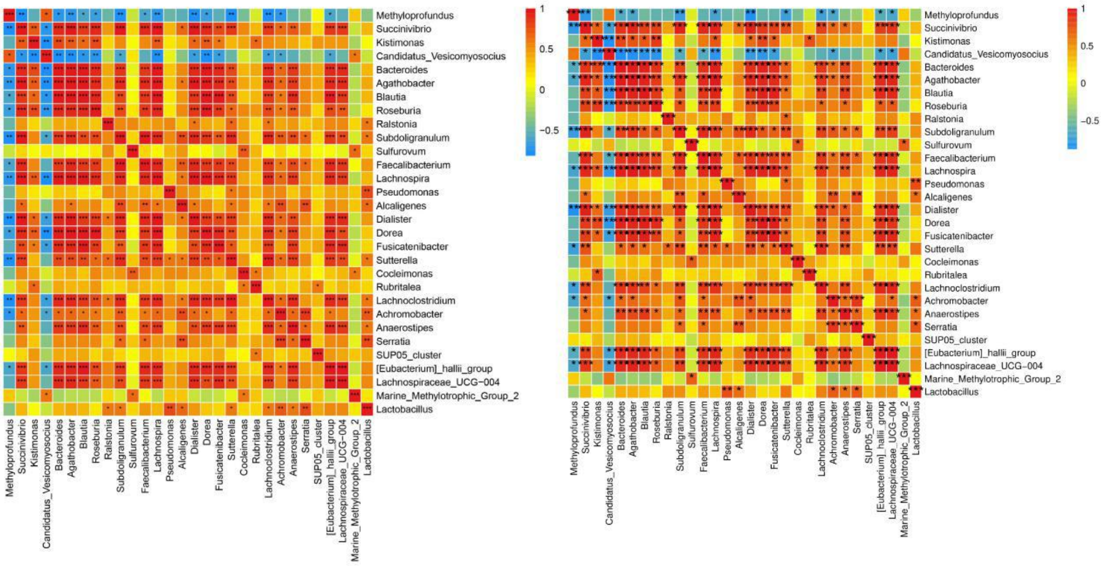

FigS13 Correlation plot depicting microbe-microbe correlations. Left: the correlation plot (with PA1 and NPA1). Right: the correlation plot (without PA1and NPA1).
