# Supplementary material for: Effects of scale worm parasitism on interactions between the symbiotic gill microbiome and gene regulation in deep sea mussel hosts
Source: Front Microbiol. 2022 Aug 15;13:940766. doi: 10.3389/fmicb.2022.940766 (PMC9421265; doi:10.3389/fmicb.2022.940766)
Supplement: Supplementary file 3 [file Data_Sheet_2.pdf]

Supplementary Figures

Fig. S1. The core microbiota shared among all samples

Fig.S2. Number of different OTUs between PA and NPA. Numbers in the overlapping regions refer to OTUs in comparison

Fig. S3. Length information of the assembled transcripts (a) and unigene (b)

Fig. S4. Characteristics of the homology search of the assembled transcriptome unigenes against the database

Fig. S5. E-value distribution of each transcriptome unigene (left). Species distribution of all homologues of the assembled transcriptome unigenes with an E-value of at least 10^{-5}(right)

Fig S6. Chart depicting the GO annotation classification. The horizontal axis represents the GO term, and the vertical axis is the number of genes annotated to the GO term.

FigS7. Statistical map of the KEGG metabolic pathway classification. The vertical axis represents the name of the KEGG metabolic pathway, and the horizontal axis shows the number of genes annotated to this pathway and their proportion to the total number of genes annotated. Genes were divided into five branches according to the KEGG metabolic pathway involved as follows: A, cellular processes; B, environmental information processing; C, genetic information processing; D, metabolism; E, organismal systems.

Fig S8 Relative bacterial abundance at family order and class level in NA and NPA.

Supplementary Tables

Table S1 Quality of sequencing of 16S rRNA

Table S2 Quality of sequencing of transcriptome

Table S3 Summary of transcriptome unigene annotation

Table S4 Length interval distribution of transcripts

Table S5 Correlation between TOP30 microbe-microbe

Table S6 Correlation between TOP30 microbe-347DEGs

Table S7 DEGs with annotated functions related to immune response, nutrients anabolism and growth-related genes

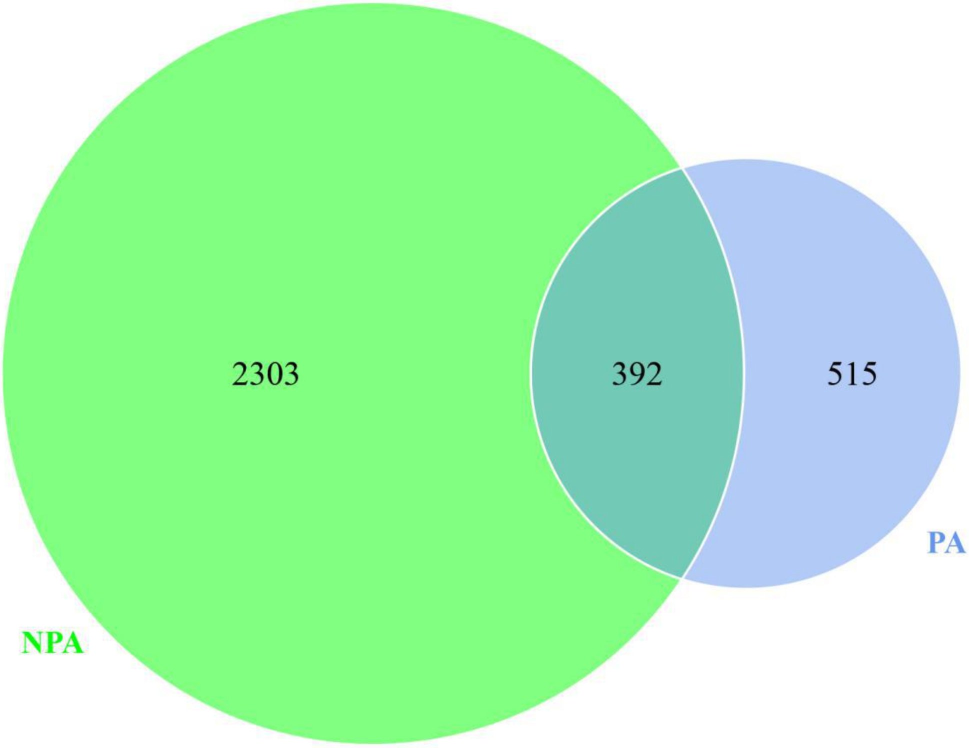

Fig.S1. Number of different OTUs between PA and NPA. Numbers in the overlapping regions refer to OTUs in comparison.

(a)

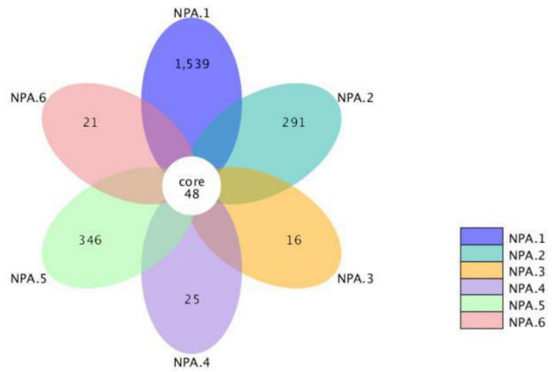

(b)

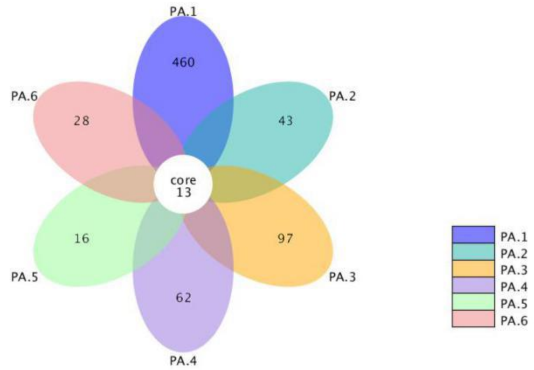

Fig. S2. The core microbiota shared among all samples.

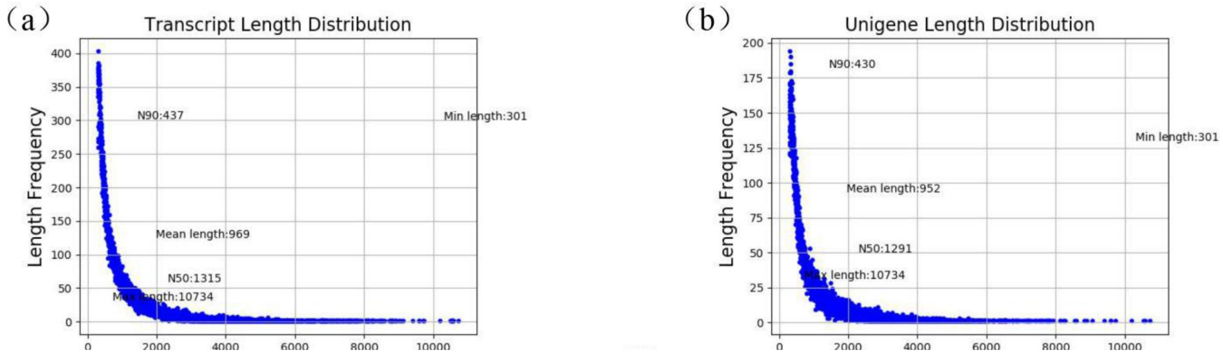

Fig. S3. Length information of the assembled transcripts (a) and unigene (b).

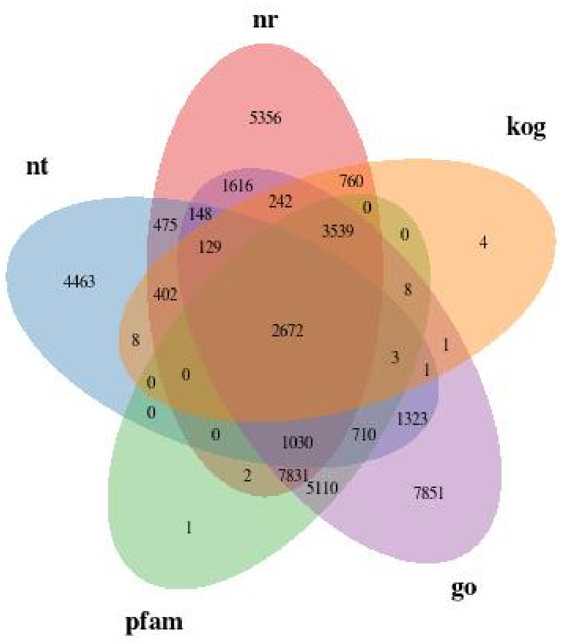

Fig. S4. Characteristics of the homology search of the assembled transcriptome unigenes against the database.

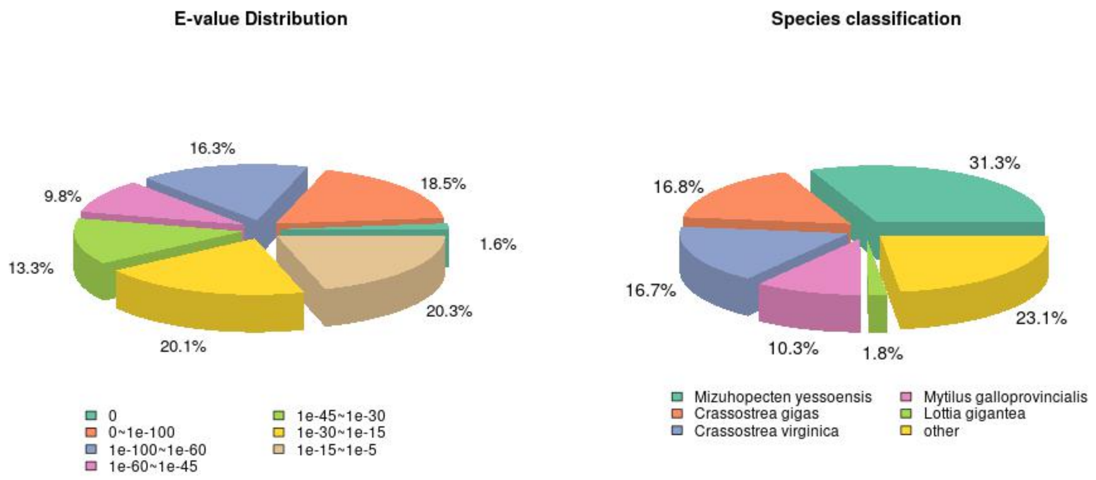

Fig. S5. E-value distribution of each transcriptome unigene (left). Species distribution of all homologues of the assembled transcriptome unigenes with an E-value of at least 10^{-5}(right).

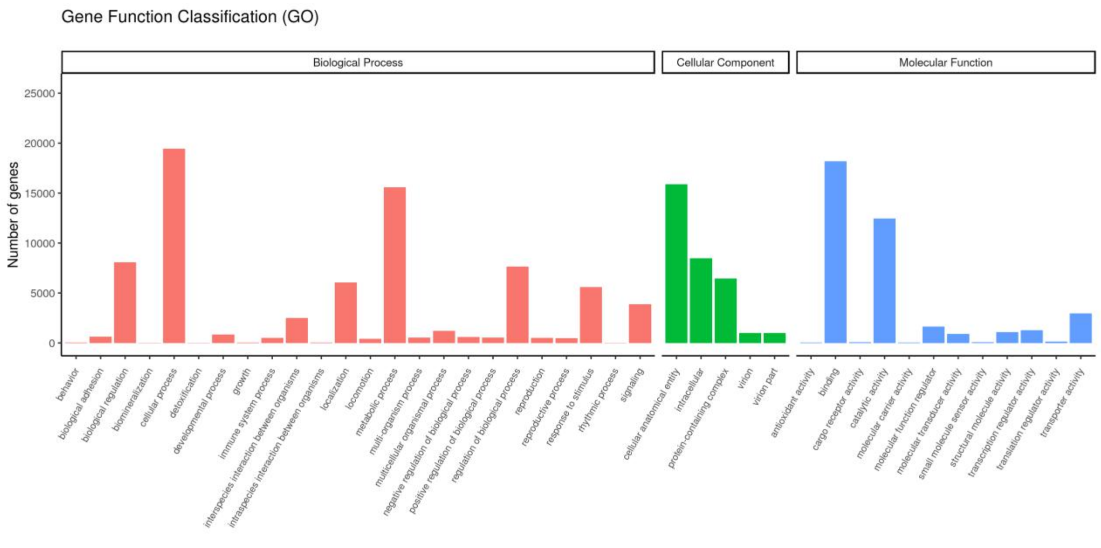

Fig S6. Chart depicting the GO annotation classification. The horizontal axis represents the GO term, and the vertical axis is the number of genes annotated to the GO term.

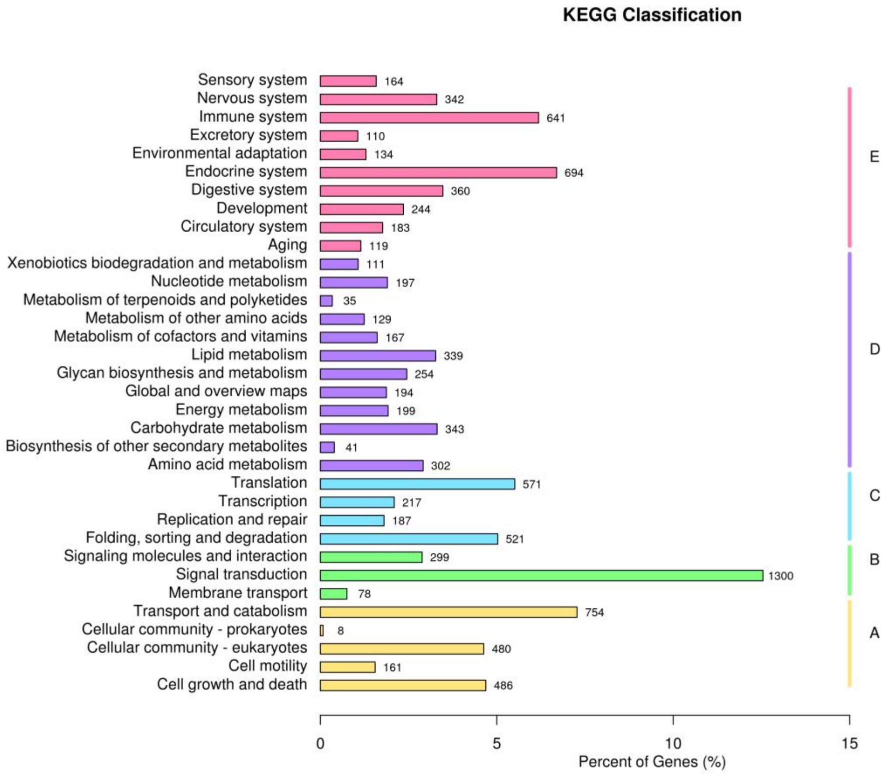

FigS7. Statistical map of the KEGG metabolic pathway classification. The vertical axis represents the name of the KEGG metabolic pathway, and the horizontal axis shows the number of genes annotated to this pathway and their proportion to the total number of genes annotated. Genes were divided into five branches according to the KEGG metabolic pathway involved as follows: A, cellular processes; B, environmental information processing; C, genetic information processing; D, metabolism; E, organismal systems.

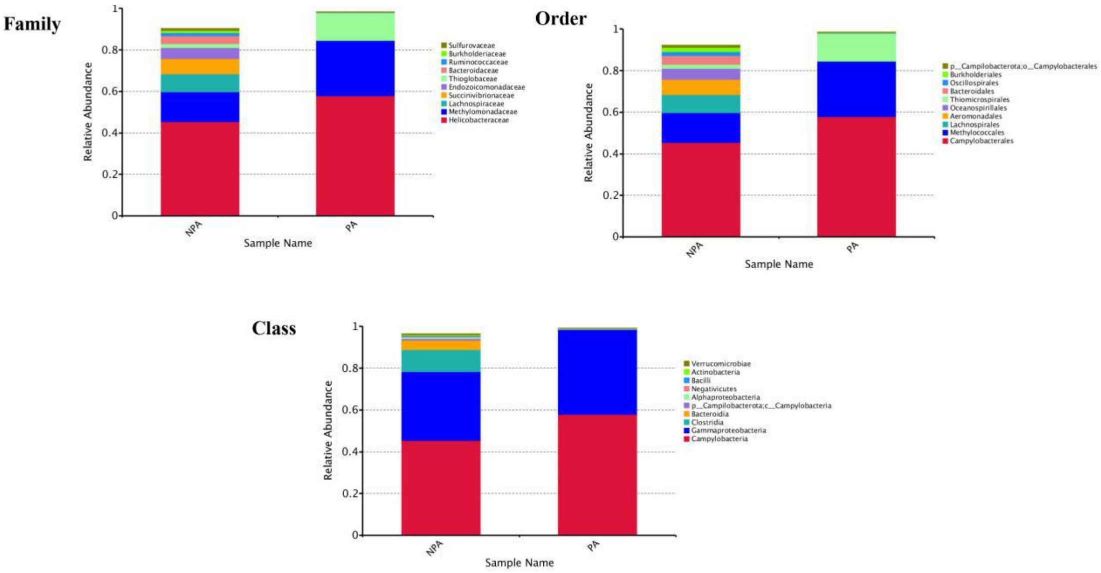

Fig S8 Relative bacterial abundance at family order and class level in NA and NPA.
